# Adverse Effects and Withdrawal Symptoms of Prolonged Glucocorticoid Therapy in Chronic Rheumatoid Arthritis and Systemic Lupus Erythematosus: A Systematic Review

**DOI:** 10.7759/cureus.99081

**Published:** 2025-12-12

**Authors:** Lincoln Xavier Naranjo Palacio, Aylin Kerime Rojas López, Bryan Nicolás Forero Vásquez, Sebastián Alejandro Ávila Alarcón, Julian Eduardo Bedoya Jaramillo, Mayra Nayeli Estrada Garcia, Rafael Eduardo Escandón González

**Affiliations:** 1 Nephrology, Valley Kidney Care, McAllen, USA; 2 Emergency/General Practice, Clinica Internacional de Traumatología, Quito, ECU; 3 Internal Medicine, Hospital General de Culiacán “Dr. Bernardo J. Gastélum”, Culiacán, MEX; 4 Internal Medicine, Universidad de Ciencias Aplicadas y Ambientales, Bogotá, COL; 5 College of Medicine, Universidad Anáhuac Cancún, Cancún, MEX; 6 Internal Medicine, Universidad San Sebastián, Puerto Montt, CHL; 7 Internal Medicine, Universidad Nacional Autónoma de México, Mexico City, MEX; 8 Internal Medicine, Centros Médicos Colsanitas, Keralty, Cali, COL

**Keywords:** drug-related side effects and adverse reactions, glucocorticoid therapy, rheumatoid arthritis, systemic lupus erythematosus (sle), withdrawal symptoms

## Abstract

The objective of this systematic review was to assess adverse effects and long-term withdrawal symptoms of prolonged glucocorticoid (GC) therapy in patients with rheumatic diseases, e.g., rheumatoid arthritis (RA) and systemic lupus erythematosus (SLE). The review synthesizes evidence from randomized controlled trials (RCTs), clinical trials, and observational cohorts in order to evaluate the safety and tolerability of long-term GC use and the effect of GC withdrawal strategies. The systematic review followed the Preferred Reporting Items for Systematic Reviews and Meta-Analyses (PRISMA) 2020 guidelines. A complete literature search on PubMed, Scopus, and Google Scholar was performed using both text words and controlled vocabulary. Only RCTs and clinical trials published in English from January 1, 2020, to October 31, 2025, were included. Only RCTs and observational cohort studies were considered. RCT's methodological quality was assessed using the Cochrane Risk of Bias 2.0 (RoB 2.0) tool and non-randomized studies by the Risk of Bias in Non-randomized Studies of Interventions (ROBINS-I) tool. ROBVIS software (University of Bristol, Bristol, United Kingdom) was used to visualize the RoB. A total of 10 studies of low to moderate RoBs were considered to synthesize evidence. In conclusion, the balance of evidence strongly supports the European Alliance of Associations for Rheumatology (EULAR) recommendations to taper and discontinue GCs whenever clinically feasible. Although there is actual danger of disease flare, the evidence suggests less long-term organ damage, particularly in SLE, and prevention of multi-system adverse events, indicating a clear clinical advantage for attempting GC withdrawal. This reinforces the significance of discontinuation as a cornerstone of standard care. The findings support a gradual process of tapering as a safer and more efficient solution than sudden withdrawal. Thus, clinicians need to take into account the individual patient factors to increase the likelihood of successful discontinuation.

## Introduction and background

Research into adverse effects and withdrawal symptoms associated with long-term glucocorticoid (GC) treatment in rheumatoid arthritis (RA) and systemic lupus erythematosus (SLE) has become a significant focus of study due to the widespread clinical use of GC in the management of inflammatory and autoimmune diseases [1]. Since their introduction in the mid-20th century, GCs have become indispensable due to their anti-inflammatory and immunosuppressant properties, but their prolonged use is associated with multiple systemic complications [2]. Epidemiological data have shown that around 1-3% of the population go through chronic GC therapy, with a rising prevalence corresponding to different diseases, including rheumatic diseases [3,4]. The scientific and clinical relevance of this problem is supported by the high rates of GC-associated osteoporosis, cardiovascular conditions, and neuropsychiatric issues that contribute to associated morbidity, healthcare burden, and impaired quality of life (QOL) [5].

Despite recognizing these adverse effects, the issue of managing GC-related complications and withdrawal symptoms remains inadequately addressed in clinical practice and academic literature [6]. Notably, GC-induced osteoporosis, cardiovascular events (e.g., major adverse cardiovascular events), and psychiatric effects (e.g., mood disorders and psychosis) are a complex challenge [7]. There is a significant lack of understanding of the best available strategies to reduce the GC tapering dose and minimize withdrawal symptoms [8]. Both the dosage-response criteria and the question of tapering GCs remain controversial when assessing benefits and risks. The current cohort studies are showing variability in reporting cardiovascular outcomes and unspecified psychiatric symptomatology [7]. This uncertainty in the current literature underscores the need to properly address these gaps, which can prevent complications and ease the disease burden on the healthcare system [9].

A conceptual framework for this review included the pharmacological side effects of GC and the physiological basis for hypothalamic-pituitary-adrenal (HPA) axis suppression [8,10]. GCs have an anti-inflammatory effect by modulating gene transcription but also have adverse effects, including bone resorption, cardiovascular changes, and dysregulation of neuropsychiatric pathways [11]. There is an inverse relationship between withdrawal and long-term exposure to doses that emphasizes the need for clinical management to address the unwanted withdrawal effects and educate patients [12]. This framework supports the systematic assessment of the adverse effects and withdrawal challenges to achieve better clinical outcomes.

The rationale for this systematic review was to thoroughly investigate the adverse effects associated with long-term GC use in chronic diseases while addressing challenges of GC withdrawal in clinical practice, academic knowledge, and patient education [6,13]. The objective of this review was to fill these gaps, enhance understanding in this area, and guide evidence-based practices for risk mitigation and patient management. The findings provided important insights to optimize the therapeutic approaches and patient safety. The objective of this systematic review was to assess the adverse effects of prolonged GC therapy in patients. The review also examined the long-term withdrawal symptoms of GC therapy in patients with rheumatic diseases (e.g., RA and SLE). The review synthesized evidence from randomized controlled trials (RCTs), clinical trials, and observational cohort studies. It also evaluated the safety and tolerability of long-term GC use. Lastly, it aimed to assess the effect of GC withdrawal in clinical practice.

## Review

Methodology

The Preferred Reporting Items for Systematic Reviews and Meta-Analyses (PRISMA) 2020 guidelines were followed for the systematic review. The Population, Intervention, Comparator, and Outcome (PICO) framework was used to develop the research question (Table 1) [14].

**Table 1:** PICO framework using text words and controlled vocabulary. RCT: randomized controlled trial; MeSH: medical subject heading; RA: rheumatoid arthritis

Concepts	Text Words	Controlled Vocabulary
Population/Problem	Adults diagnosed with RA and systemic lupus erythematosus	"Adult" [MeSH], "Arthritis, Rheumatoid" [Mesh], "Lupus Erythematosus, Systemic" [MeSH]
Intervention/Exposure	Glucocorticoid therapy (e.g., prednisone)	"Glucocorticoids" [MeSH]
Comparative	Placebo or glucocorticoid withdrawal strategies (e.g., rapid tapering vs. slow tapering)	“Withdrawal Strategies”
Outcomes	Adverse effects such as osteoporosis, fractures, musculoskeletal effects, psychiatric effects, skin effects, withdrawal symptoms (steroid withdrawal syndrome, disease flare-ups in RA), and functional status during and after withdrawal.	"Functional Status" [MeSH], "Symptom Flare Up" [MeSH], "Steroid Withdrawal Syndrome"
Study Design	RCT, Clinical trials, Cohort Studies, Observational Studies	"Randomized Controlled Trial" [Publication Type]

Research Question

In adults diagnosed with RA and SLE, what are the efficacy and adverse effects of long-term GC treatment? How can GC withdrawal be achieved (e.g., rapid tapering vs. slow tapering) effectively?

Search Strategy

The search strings used in three electronic databases, such as PubMed, Scopus, and Google Scholar, are given in Table 2. Papers written in English and published from January 1, 2020, to October 31, 2025, were included.

**Table 2:** Search strings used on selected databases. MeSH: medical subject heading

Database	Search Strings
PubMed	("Rheumatoid Arthritis"[MeSH] OR "Systemic Lupus Erythematosus"[MeSH]) AND ("Glucocorticoids"[MeSH] OR "Prednisone" OR "Prednisolone" OR "Methylprednisolone" OR "Dexamethasone" OR "Hydrocortisone") AND ("Adverse Effects"[MeSH] OR "Side Effects" OR "Toxicity" OR "Complications") AND ("Withdrawal Syndrome" OR "Adrenal Insufficiency" OR "Glucocorticoid Tapering" OR "Steroid Withdrawal" OR "Disease Reactivation") AND ("Clinical Trial"[Publication Type] OR "Randomized Controlled Trial"[Publication Type])
Scopus	("Rheumatoid Arthritis" OR "Systemic Lupus Erythematosus") ("Glucocorticoids" OR "Prednisone" OR "Prednisolone" OR "Methylprednisolone" OR "Dexamethasone" OR "Hydrocortisone") AND ("Adverse Effects" OR "Side Effects" OR "Complications" OR "Toxicities" OR "Withdrawal Symptoms" OR "Steroid Withdrawal Syndrome") AND ("Randomized Controlled Trial" OR "Clinical Trial")
Google Scholar	"Rheumatoid Arthritis" OR "Systemic Lupus Erythematosus" AND "Glucocorticoids" OR "Prednisone" OR "Prednisolone" OR "Methylprednisolone" OR "Dexamethasone" OR "Hydrocortisone" AND "Adverse Effects" OR "Side Effects" OR "Complications" OR "Steroid Withdrawal" OR "Withdrawal Syndrome" AND "Randomized Controlled Trial" OR "Clinical Trial"

Inclusion Criteria

Clinical trials, RCTs, and observational cohort studies were included. Studies on adults over 18 with chronic RA and SLE were included. Long-term GC use (e.g., prednisone) was considered. Other treatments included GC tapering regimens. GC withdrawal syndrome and adverse effects (osteoporosis, hypertension, infections, etc.) studies were considered.

Exclusion Criteria

The review excluded case reports, case series, conference abstracts, editorials, letters, review papers, and meta-analyses. Individuals below 19 and animal studies were excluded. Studies with restricted or incomplete data and outcomes were not considered. Studies published before January 1, 2020, were omitted.

Study Selection Process

The studies were initially selected by filtering papers based on titles and abstracts. In the second step, the full texts of the articles were screened against the inclusion criteria. The entire process was carried out independently by the authors. The third reviewer was consulted to resolve conflicts during screening and selecting articles [15].

Methodological Quality Assessment

The review assessed RCTs using the Cochrane Risk of Bias (RoB) 2.0 tool and non-RCTs using the Risk of Bias in Non-randomized Studies of Interventions (ROBINS-I) tool. The studies were categorized as low RoB, unclear/some concerns RoB, and high RoB [16,17]. The ROBVIS software (University of Bristol, Bristol, United Kingdom) was used to visualize the RoB traffic light and summary plots to illustrate the quality assessment of each included study [18].

Data Extraction and Synthesis

The data was extracted using Excel 365 (Microsoft Corp., Redmond, WA, USA) [19]. After selecting studies, independent reviewers collected data in parallel to ensure interpretation uniformity and minimize variability. The data extracted from each paper were author, year, study design (RCT, clinical trial, observational cohort), and sample size.

The following detailed data were independently extracted by the reviewers, including population characteristics, disease type (condition), age, percentage of females, medication dosage to control GCs, and tapering strategy for medication dosage and frequency. Intervention details included the type of GC, dose, treatment duration, and taper regimen. The outcomes, such as adverse effects (osteoporosis, infections, psychiatric effects, etc.) and withdrawal symptoms (adrenal insufficiency, disease reactivation, withdrawal syndrome), were separately extracted and analyzed. Cases of adverse effects and their intensity, rate of successful discontinuation of GCs, acute episodes, and functional condition of illnesses were tabulated.

The evidence was synthesized using a narrative approach. (1) Preliminary coding: Two reviewers coded quantitative and qualitative data (adverse effects and withdrawal symptoms) independently in preliminary categories of outcomes. (2) Theme development: These early codes created these broad themes. The final evidence was narrated and presented in comparative tables to make it easy to understand across research and highlight comparable trends. The synthesis of the theme meaningfully integrated to support clinically relevant conclusions.

Results

Selection Process of Studies

**Figure 1:**
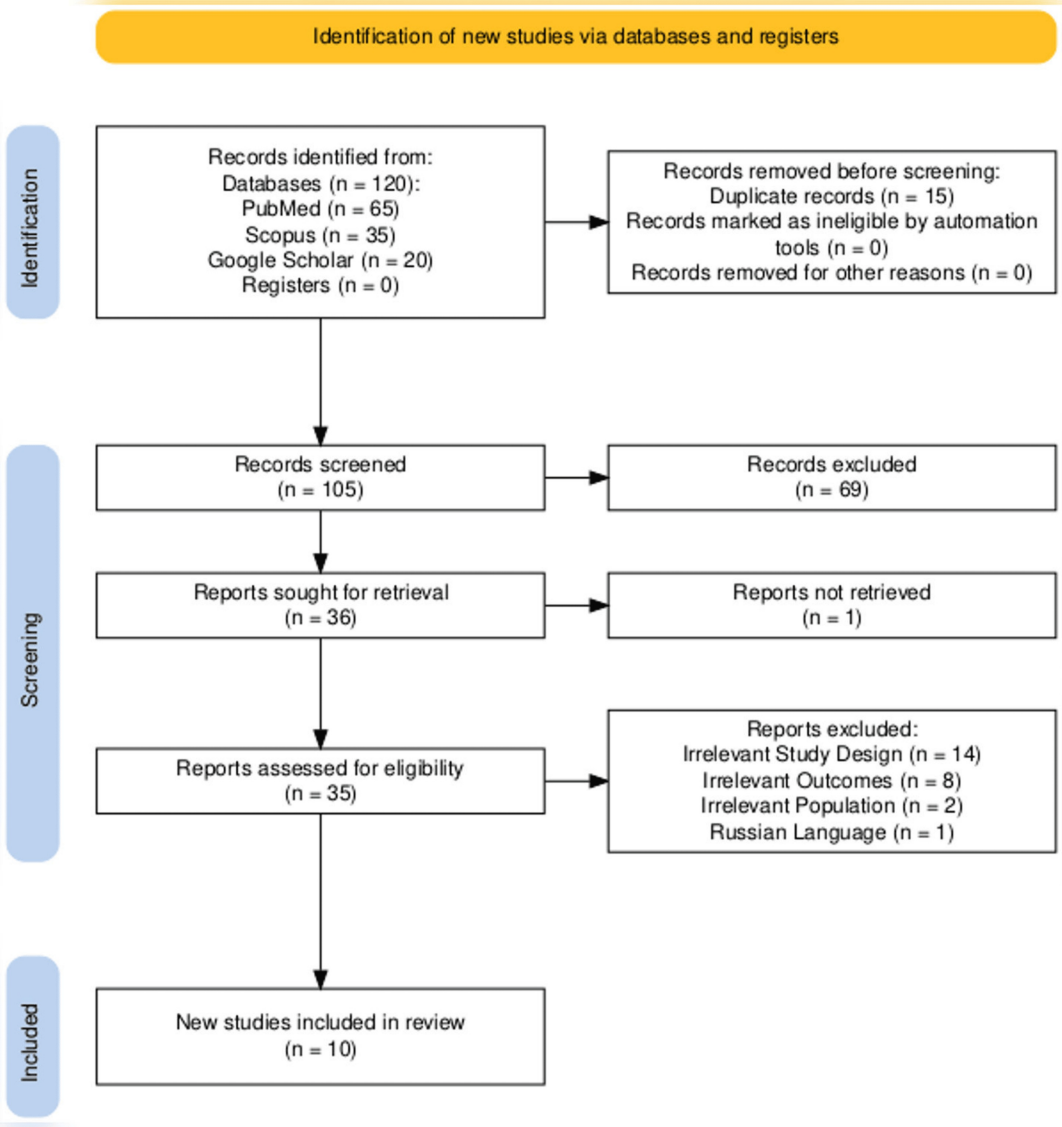
PRISMA flow chart. PRISMA: Preferred Reporting Items for Systematic Reviews and Meta-Analyses

The initial database search identified 120 papers, of which 15 duplicates were removed using EndNote version 20.1 (Clarivate, London, UK). After evaluating the titles and abstracts of the remaining 105 papers, 69 were excluded as irrelevant. One full-text article could not be retrieved. The eligibility of the remaining 35 studies was then fully assessed. Figure 1 shows the reasons for removing 25 studies during full-text screening. Ultimately, 10 studies were included for quality assessment, with their characteristics and key findings presented in Tables 3-4.

**Table 3:** Characteristics of the included studies. RA: rheumatoid arthritis, SLE: systemic lupus erythematosus, GC: glucocorticoid, DAS28: disease activity score 28 joints, ESR: erythrocyte sedimentation rate, EULAR: European Alliance of Associations for Rheumatology, mSACQ: modified serologically active clinically quiescent, SELENA-SLEDAI: safety of estrogen in lupus erythematosus national assessment-systemic lupus erythematosus disease activity index, b/tsDMARDs: biologic/targeted synthetic disease-modifying anti-rheumatic drugs, GLORIA: glucocorticoid reduction in rheumatoid arthritis, GULP: glucocorticoid use in newly diagnosed SLE patients, UPA: upadacitinib, ADA: adalimumab, NA: not applicable

Author (Year)	Study Design	Data Source/Centre	Sample Age/Percentage of Females	Condition	Intervention/Exposure	Tapering Strategy/Discontinuation Strategy	Primary Outcome
Ruyssen-Witrand et al. (2025) [20]	STAR (RCT)	France	102/62.4 years (SD 13.4)/70.6%	RA patients who were in low disease activity (LDA)	20 mg/day of hydrocortisone for three months, then 10 mg/day for three months, followed by discontinuation. This group also received a placebo of prednisone with a tapering scheme identical to the prednisone tapering group.	Patients followed a tapering scheme of prednisone over four months, starting with 4 mg/day for one month, then 3 mg/day for one month, 2 mg/day for one month, and finally 1 mg/day for one month. This group also received a placebo of hydrocortisone.	Percentage of patients achieving GC discontinuation at 12 months.
Katz et al. (2024) [21]	Longitudinal (LUPUS) cohort study	USA	LOS = 6839, 49.7 ± 12.7 years/92.3%; FORWARD = 5220, 58.5 ± 13.0 years/94.1%	SLE	The study examined patterns of GC use, including dosage (0, 1-5, 5-7.5, and ≥7.5 mg/day).	NA	The study investigated the association between GC exposure and the occurrence of adverse health conditions (AHCs) and health care use.
Katsumata et al. (2024) [22]	Multinational observational cohort study	Asia Pacific Lupus Collaboration (APLC) patient cohort, which includes patients recruited from 24 sites across 12 countries	1850/40.0 years (SD 13.5)/91.6% women	mSACQ SLE patients.	≤7.5 mg/day of prednisolone-equivalent GCs.	Tapering GCs by 1 mg/day of prednisolone-equivalent	Disease flare (including mild-to-moderate and severe flares) according to the SELENA-SLEDAI flare index definitions.
Spinelli et al. (2023) [23]	Observational cohort open-label study	Italy	30/60 ± 13 years/86.7%	RA	Tofacitinib treatment: Patients started treatment with tofacitinib (5 mg twice/day) while maintaining a stable dose of prednisone for the first four weeks	Predetermined schedule: GCs were tapered according to a predetermined schedule until complete discontinuation at week 12, specifically for patients who achieved at least a moderate EULAR response (decrease of >1.2 in DAS28_CRP) after four weeks of tofacitinib treatment.	The primary endpoint was the percentage of patients discontinuing GCs after 12 weeks of tofacitinib treatment.
Giollo et al. (2023) [24]	Observational, monocentric, prospective cohort study	Italy	229/57 ± 13 years/82.1%	RA	b/tsDMARDs: (87%) Very-low-dose GCs: (<5 mg/day)	Persistent GC discontinuation was defined as GC therapy not being prescribed for more than two successive visits (six months apart).	The primary outcome was the percentage of patients who discontinued GC due to persistent GC.
Almayali et al. (2023) [25]	Follow-up of the GLORIA randomised placebo-controlled trial	European Dutch Countries	191 flare analysis/155 patients primary outcome analysis of DAS28 and adrenal insufficiency/72 (5) years/69%	RA	Prednisolone 5 mg/day or placebo over three months.	The tapering strategy involved a linear reduction of prednisolone 5 mg/day or placebo to zero over three months. The schedule specified taking one capsule (representing the study medication) on certain days and zero on others, progressively decreasing the frequency of intake.	The primary outcome was the three-month change in DAS28-ESR (Disease Activity Score 28 joints - Erythrocyte Sedimentation Rate) after tapering, starting from the final trial visit.
Floris et al. (2022) [26]	Prospective observational (GULP) cohort study	UK	127/36.7 years (± 13.4)/82.7%	GULP	Prednisone (PDN) with baseline daily doses categorized as low (<40% of patients), moderate (51.2%), and high (17.3%).	Concomitant treatment: All patients received hydroxychloroquine (HCQ).	The study aimed to investigate the feasibility of tapering PDN below 5 mg/day and its associated short-term outcomes.
Fleischmann et al. (2022) [27]	SELECT-COMPARE (RCT)	USA/UK/EU	1,629/38.7 years (± 13.4)/85.7%	RA	Upadacitinib (UPA): Patients received UPA 15 mg once daily	Adalimumab (ADA): Patients received ADA 40mg every other week; Background GCs could be tapered or discontinued starting at week 26 per the physician's discretion.	The study described the patterns of GC discontinuation.
Boers et al. (2022) [28]	Multicenter, open-label (GLORIA trial) observational cohort study	Norway	449/72-88 years/70%	RA	Patients received prednisolone at a dose of 5 mg/day	Placebo	The primary benefit outcomes included improvement in disease activity, measured by DAS28, and joint damage progression, measured by Sharp/van der Heijde scores.
Tselios et al. (2021) [29]	Prospective cohort study	Canada	204 patients Maintenance group = 102, 44.1 ± 15.4 years, 90.2%; Withdrawal group = 102, 41.7 ± 12.9 years, 87.3%	SLE	Withdrawal group: Gradually tapered prednisone within two years; Maintenance group: Maintained a low prednisone dose (5 mg/day)	Gradual tapering: Prednisone dose was reduced by 1 mg/day over seven weeks.	Clinical flares (any increase in clinical SLEDAI-2K, increase in SLEDAI-2K with treatment escalation, or increase of ≥4 in clinical SLEDAI-2K) and damage accrual (Systemic Lupus International Collaborating Clinics Damage Index-SDI).

**Table 4:** Key findings of the included studies. GCRA: rheumatoid arthritis, SLE: systemic lupus erythematosus, GC: glucocorticoid, DAS28: disease activity score 28 joints, ESR: erythrocyte sedimentation rate, EULAR: European Alliance of Associations for Rheumatology, mSACQ: modified serologically active clinically quiescent, SELENA-SLEDAI: safety of estrogen in lupus erythematosus national assessment: systemic lupus erythematosus disease activity index, b/tsDMARDs: biologic/targeted synthetic disease-modifying anti-rheumatic drugs, GLORIA: glucocorticoid reduction in rheumatoid arthritis, GULP: glucocorticoid use in newly diagnosed SLE patients, UPA: upadacitinib, ADA: adalimumab, RARA: rheumatoid arthritis, SLE: systemic lupus erythematosus, GC: glucocorticoid, DAS28: disease activity score 28 joints, aOR: adjusted odds ratio, CI: confidence interval, ACTH: adrenocorticotropic hormone, CDAI: clinical disease activity Index, DAS28-CRP: disease activity score 28 joints-C-reactive protein, ECLAM: european consensus lupus activity measure, PDN: Prednisone, IRR: incidence rate ratio, SDI: systemic lupus international collaborating clinics damage index, HR: hazard ratio, ACTH/cortisol: adrenocorticotropic hormone/cortisol, NA: not applicable

Author (Year)	Adverse Effects	Withdrawal Symptoms	Key Findings	Conclusion
Ruyssen-Witrand et al. (2025) [20]	Hydrocortisone replacement group = 43 patients (81%). Prednisone tapering group = 33 patients (67%).	After dose reduction, GC withdrawal symptoms include depression, lethargy, weakness, nausea, and arthralgias. GC withdrawal after long-term exposure might cause depression or confusion. Withdrawal symptoms are identical between groups.	At 12 months, 55% of patients in the hydrocortisone replacement group and 47% in the prednisone tapering group achieved GC discontinuation (p=0.4)	So, no significant difference between the two strategies. No cases of acute adrenal insufficiency were observed.
Katz et al. (2024) [21]	LOS Cohort: Individuals using GCs were more likely to report osteoporosis (adjusted odds ratio (aOR) 1.7, 95% confidence interval (CI) 1.2-2.6) and cataracts (aOR 1.6, 95% CI 1.04-2.6) at baseline. High doses of GCs were associated with a greater incidence of osteoporosis, fractures, and cataracts. FORWARD Cohort: Individuals using GCs were more likely to report diabetes (aOR 5.1, 95% CI 2.2-12.0), osteoporosis (aOR 4.5, 95% CI 2.6-8.0), and fractures (aOR 6.5, 95% CI 3.8-11.1) at baseline. A significant difference in infection incidence was noted.	The study does not explicitly mention withdrawal symptoms. It focuses on adverse health conditions and healthcare utilization associated with ongoing GC use.	GC use was relatively consistent over the 15-year study period (2006-2021), despite recommendations for steroid-sparing. A significant portion of individuals with SLE remained on steroids, often at high doses, leading to a substantial health and healthcare burden.	The analyses provide additional evidence of the potential health and healthcare burden associated with GC use, underscoring the need for other effective treatments for individuals with SLE.
Katsumata et al. (2024) [22]	NA	NA	Tapering GCs by 1 mg/day of prednisolone was not associated with an increased risk of overall or severe flare. The adjusted hazard ratios (HRs) were 1.02 (95% CI, 0.99 to 1.05; p=0.27) for overall flare and 0.98 (95% CI, 0.96 to 1.004; p=0.11) for severe flare in patients receiving 0-7.5 mg/day of GCs.	Cautious GC tapering is feasible and can reduce the GC burden without increasing flare risk.
Spinelli et al. (2023) [23]	Tofacitinib Discontinuation: During the 48-week study, three patients discontinued tofacitinib due to adverse events, including one for endometrial malignancy, one for severe infection, and one for paresthesia. Special Interest Adverse Events: No cases of varicella zoster reactivation or major cardiovascular events (MACE) or thromboembolic events were recorded.	NA	Nine out of 30 patients (30%) achieved complete discontinuation of prednisone at week 12. By week 24, an additional 12 patients (46%) withdrew GCs, and by week 48, 12 out of 30 patients (40%) had discontinued prednisone.	Hence, approximately one-third of patients achieved discontinuation of GCs, with significant reductions in daily doses, supporting the 2022 EULAR recommendations for tapering GCs as a bridging therapy.
Giollo et al. (2023) [24]	Cardiovascular Morbidity: Use of very-low-dose GCs was associated with hypertension (20% vs. 11%) and myocardial infarction (2.3% vs. 0%). Patients who stopped taking very-low-dose GCs reported fewer adverse effects than those who continued taking them. Other Comorbidities: Patients who continued GCs had higher rates of cardiovascular disease (29% vs. 21%) and hypertension (20% vs. 11%) compared to those who stopped. No cases of peptic ulcer disease or stroke were reported.	NA	A substantial proportion of RA patients treated with b/tsDMARDs continue to receive very-low-dose GCs without significantly improving disease control. Failure to discontinue GC therapy was associated with persistent moderate disease activity. Cardiovascular Risk: Persistent low-dose GC therapy was hypothesized to be related to cardiovascular disease.	While very-low-dose GC use is common in RA patients on b/tsDMARDs, it often fails to improve disease control significantly and is associated with increased cardiovascular risks. Shorter disease duration at the start of b/tsDMARDs was a key factor in successful GC cessation, emphasizing the challenge of discontinuing GCs in long-standing RA.
Almayali et al. (2023) [25]	Disease Flares: 45% of prednisolone patients compared with 33% in the placebo group, (RR) 1.37, 95% CI 0.95 to 1.98; p = 0.12).	Adrenal Insufficiency: No evidence of adrenal insufficiency was found based on signs, symptoms, or ACTH/cortisol spot measurements.	Increased Disease Activity: Tapering prednisolone moderately increased RA disease activity to levels similar to those in the placebo group, though mean disease activity remained low. Feasibility and Safety: Withdrawal of low-dose prednisolone in a three-month schedule was found to be feasible and safe after two years of administration, with no evidence of adrenal insufficiency.	Tapering and stopping prednisolone 5 mg/day over three months in senior RA patients (aged 65+) who had been on treatment for two years moderately increased RA disease activity to levels observed in the placebo group, while maintaining low disease activity. Despite a numerical increase in flare risk, there was no evidence of adrenal insufficiency, suggesting that this withdrawal strategy is feasible and safe.
Floris et al. (2022) [26]	GC-Related Damage: Never tapering PDN <5 mg/day was associated with a higher risk of developing GC-related damage (OR 5.9; p=0.014). Disease Relapse: The disease relapse rate did not statistically differ between patients tapering PDN <5 mg/day (42.4%) and those tapering PDN without dropping below 5 mg/day (46.4%).	NA	Seventy-three patients (57.5%) successfully tapered and maintained PDN <5 mg/day, while 17 patients (13.4%) discontinued PDN within a two-year follow-up.	Tapering PDN below 5 mg/day was achieved and maintained in about half of newly diagnosed SLE patients. Spending every month on PDN <5 mg/day was associated with lower damage accrual (IRR: 0.96; p=0.007). Predictors of Failed Tapering: Active renal involvement (HR: 0.41; p=0.009) and lower C3 serum levels (HR: 1.04; p=0.025) were associated with a lack of PDN tapering below 5 mg/day. Disease Activity and Dose Increase: High ECLAM scores were associated with a greater probability of increasing PDN dose (OR: 1.6; p=0.004).
Fleischmann et al. (2022) [27]	Disease Worsening: Few patients receiving UPA experienced worsening of disease following GC discontinuation (1% CDAI increase >2; 7% DAS28-CRP increase >0.6), and none on ADA experienced worsening. GC Reintroduction: GCs were reintroduced in 14% of patients on UPA and 19% on ADA. Serious Infection Rates: Rates of severe infection before and after GC discontinuation were 0.8 (95% CI 0.0-4.2) and 1.5 (0.2-5.4) events per 100 patient-years for UPA and 7.7 (1.6-22.4) and 0 E/100 PY for ADA, respectively.	NA	Disease Control Maintenance: In patients who achieved disease control and successfully discontinued GCs, disease control was maintained in almost all without worsening disease activity over time. Higher Disease Control with UPA: At the time of GC discontinuation, a numerically higher proportion of UPA-treated patients were in disease control compared to ADA-treated patients (e.g., CDAI ≤2.8: 55% vs. 32%, DAS28-CRP <2.6: 71% vs. 48%).	In RA patients who achieved disease control and subsequently discontinued GCs, disease control was largely maintained over time without worsening disease activity.
Boers et al. (2022) [28]	Harm Outcome Incidence: 60% of prednisolone patients versus 49% of placebo patients experienced the harm outcome, resulting in an adjusted relative risk (RR) of 1.24 (95% CL 1.04, p = 0.02). The number needed to harm was 9.5. Specific Adverse Events: The difference in adverse events was most marked for infections, which were primarily mild or moderately severe. Other GC-specific AESIs were rare and showed no relevant differences. Mortality: During the study, one patient in the prednisolone group and two in the placebo group died. Within five months of discontinuation, three patients in the prednisolone group and 0 in the placebo group died.	NA	Prednisolone led to a significant and rapid decline in disease activity, which stabilized after one year. The adjusted mean difference in DAS28 over two years was 0.37 (95% CL 0.23, p < 0.0001). Joint damage progression over two years was significantly lower in the prednisolone group (mean 0.6 vs. 1.8 score points on placebo, difference 1.2, 95% CL 0.2, p=0.02).	Adding low-dose prednisolone to standard treatment in senior RA patients offers beneficial long-term effects on disease activity and damage progression. This benefit comes with a trade-off: a 24% increase in mostly mild-to-moderate adverse events, suggesting a favorable balance of benefit and harm.
Tselios et al. (2021) [29]	Damage accrual was less frequent in the withdrawal group (6.9% vs. 17.6%; P = 0.022). GC-dependent damage was also less in the withdrawal group (2.9% vs. 11.8%; P = 0.02)	Persistent withdrawal symptoms, but did not explicitly elaborate.	Flares: Flare rate (any increase in clinical SLEDAI-2K) was lower in the withdrawal group at both 12 months (17.6% vs 29.4%; P = 0.023) and 24 months (33.3% vs 50%; P = 0.01). Moderate to severe flares were less frequent at 24 months (14.7% vs 27.5%; P = 0.024). Damage Accrual: Damage accrual was less frequent in the withdrawal group (6.9% vs. 17.6%; P = 0.022)	Gradual GC withdrawal is safe in clinically quiescent SLE and is associated with fewer clinical flares and less damage accrual at 24 months. The study suggests that gradual withdrawal is safer than abrupt discontinuation and could be attempted in patients with clinically quiescent SLE.

Methodological Quality (RoB Assessment)

**Figure 2:**
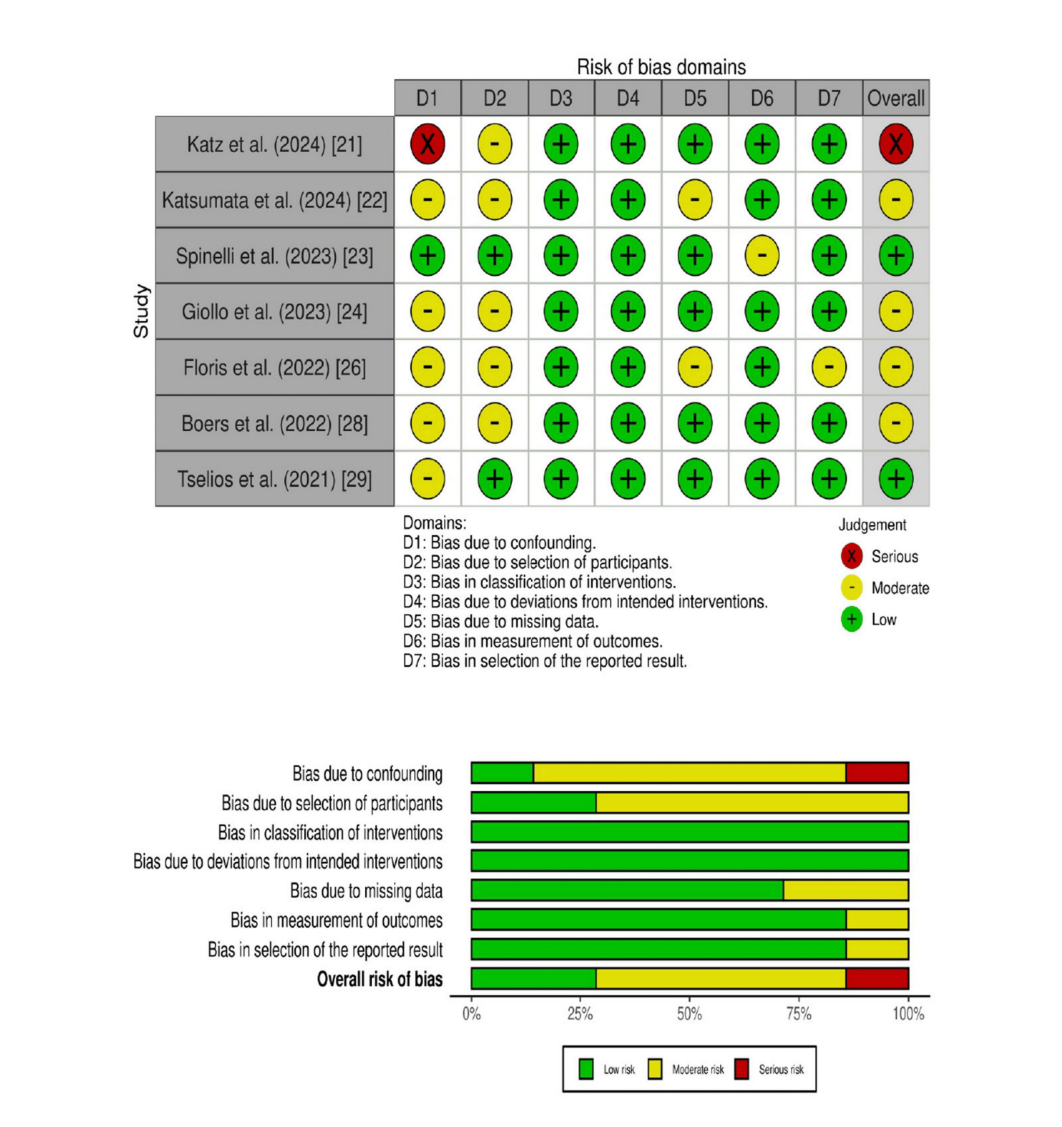
ROBINS-I assessment of quality for non-RCTs. ROBINS-I: Risk of Bias in Non-randomized Studies of Interventions; RCT: randomized controlled trial References [21-24,26,28,29]

The ROBINS-I tool's seven domains were used to assess RoB in non-randomized studies. Four out of seven studies exhibited moderate RoB due to confounding variables and bias in the selection of participants. However, two out of seven studies reported low RoB. Only one study reported serious RoB. In all studies, low RoB was reported in only the D3 and D4 domains. Overall, the RoB is low to moderate (Figure 2).

**Figure 3:**
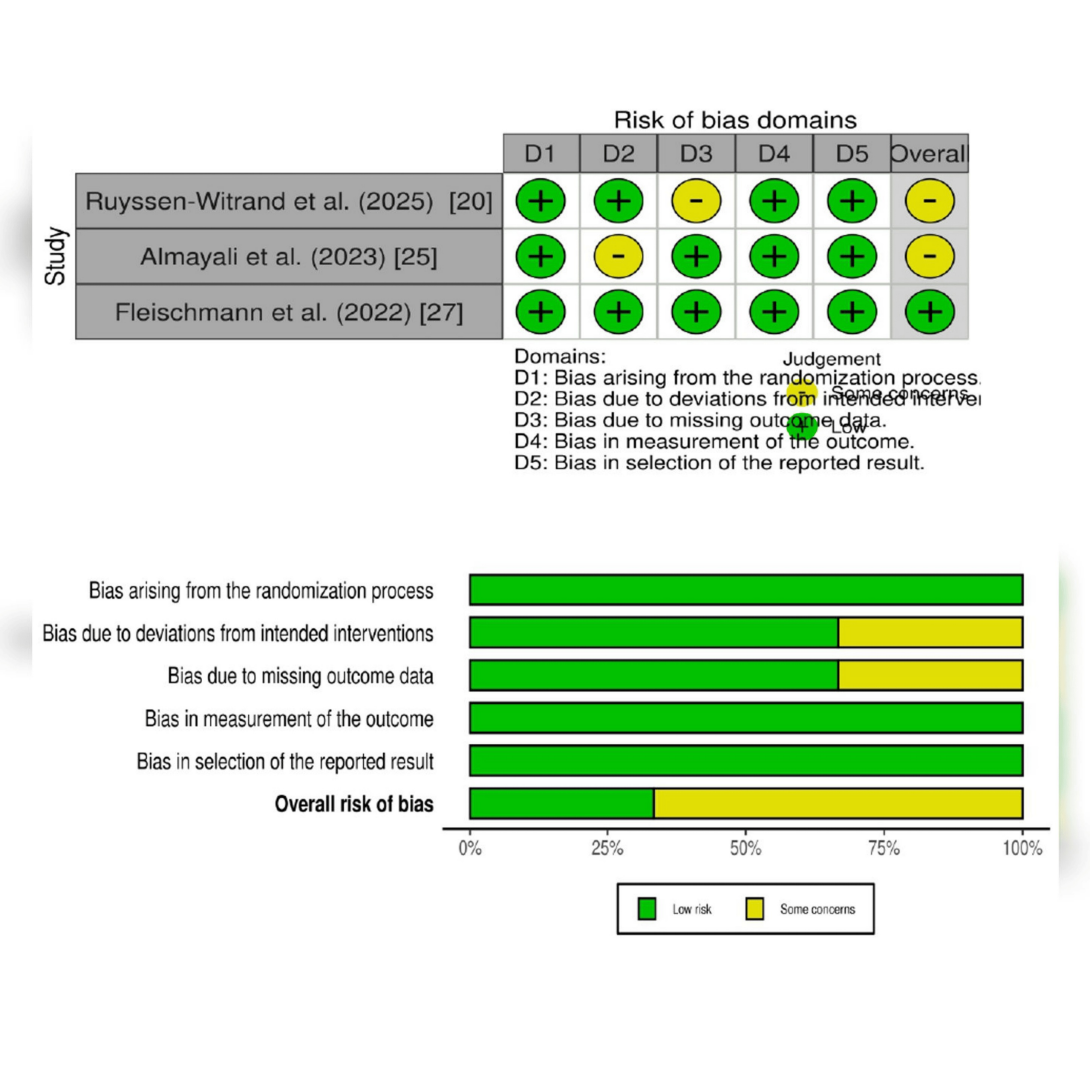
Methodological quality assessment of included RCTs using the Cochrane Risk of Bias 2.0. RCT: randomized controlled trial References [20,25,27]

The Cochrane RoB 2.0 tool's five domains were used to assess RoB in three RCTs. One RCT reported low RoB, and two had some concerns/unclear RoB. All studies exhibited moderate to low RoB (Figure 3).

Discussion

The evidence synthesized here provides a coherent picture of the GC dilemma. Adverse effects of GCs are clearly tied to long-term use, regardless of dose under 5 mg/day. Data from large cohorts indicate increased risks of cardiovascular issues, such as high blood pressure and heart attack; musculoskeletal problems like osteoporosis and fractures; and increased susceptibility to infections. This builds a strong, well-evidenced case against indefinite maintenance therapy, making GC withdrawal a high-priority therapeutic objective. Yet, challenges remain: discontinuation success is variable (about half of patients) and is influenced by age and disease duration. Flares are the most common cause of taper failure, but data show this risk may be preventable. Tapering appears safe and is associated with fewer flares and, crucially, less irreversible organ damage, especially when done gradually in quiescent SLE. While biochemical adrenal insufficiency may persist after tapering, the risk of adverse effects is reduced [20-30].

Adverse Side Effects and Withdrawal Symptoms of GC Therapy in Rheumatic Diseases: Synthesis of Evidence on the GC Dilemma in Rheumatology

GCs are a paradox in contemporary rheumatology. They have been used for more than 70 years to treat inflammatory rheumatic diseases, such as RA and SLE, due to their proven anti-inflammatory effects. However, chronic use is known to cause significant morbidity, potentially leading to comorbidities as considerable as the diseases themselves. This report synthesizes recent clinical and observational studies to provide a qualitative overview of adverse effects from maintenance GC therapy and clinical outcomes after discontinuation [26-28].

Despite new therapies like biologic and targeted synthetic disease-modifying antirheumatic drugs (bDMARDs and tsDMARDs), many patients continue long-term GC use, highlighting clinicians' ongoing challenge in balancing efficacy and safety. To address this dilemma, we will first outline the documented adverse effects of long-term GC use and then examine the challenges of tapering and discontinuation [29].

Side Effects of Maintenance GC Therapy

Long-term GC therapy requires strategic insight into the safety profile to inform clinical decision-making when administering low or very-low doses (e.g., <5 mg/day prednisone). Recent findings support the idea that prolonged exposures to GCs may lead to multi-systemic adverse events. It summarizes and compiles information on specific comorbidities of GCs observed in recent cohorts of RA and SLE patients and estimates the risks of maintenance therapy [25,27,30].

Cardiovascular Complications

The association between GC use and cardiometabolic risk is well established, and several studies have shown a strong association, even at doses considered reasonably safe. There is a high probability of having hypertension in users of GCs, which is observed in several cohorts and dosage levels. The development of hypertension in the RA cohort study, which observed high-dose GC maintenance therapy users, occurred in 19% of users compared with 9% of non-users [25]. These findings are consistent with another prospective study that analyzed very low doses (<5 mg/day) and also had an association with hypertension (20% in GC users vs. 11% in non-users) [31]. In a separate analysis about RA patients using long-term GCs (over six months), it was established that hypertension was a much more common diagnosis when compared with patients who were not using GCs [24]. The withdrawal of GCs seems to reduce cardiovascular risk. Coronary artery disease was found to be 0% in patients who discontinued GCs, compared with 4.6% in those who continued treatment [25]. Giollo et al. (2025) also examined that very-low-dose GC treatment in RA identified the occurrence of myocardial infarction as 2.3x in GC users as compared to non-users in a three-year time span [30].

Musculoskeletal Complications

One of the main adverse effects is the musculoskeletal complications induced by GCs, especially in the bone. Osteoporosis ranks among the most common comorbidities found in people taking long-term GCs. The large cohort SLE study indicates that patients undergoing GCs were much more frequently on top of osteoporosis and fracture at baseline. The risk is dose-related; the LOS cohort data showed that participants on higher doses (7.5 mg/day) were more likely to have osteoporosis and fractures during follow-up [21].

Comorbidities and Infections

In addition to cardiometabolic and musculoskeletal effects, there is an increased risk of acquiring infection and other system-related complications with the long-term use of GCs. In older patients with RA studied in the GLORIA trial, taking prednisolone 5 mg/day reported that adverse events were mostly mild to moderate and necessitated treatment [29]. Equally, the FORWARD cohort revealed a much higher infection rate among GC users than among non-users [21]. Other important comorbidities have been reported to have a chronic association with GC use. The examination of more than 700 patients on long-term GCs revealed that the prevalence of conditions such as tuberculosis, chronic kidney disease, and cataracts was much higher among long-term GC patients than among GC-naive patients [24]. The aggregate of evidence points clearly to the fact that keeping a patient on GC therapy has a high risk of the development of multi-organ morbidity.

GC Withdrawal

One of the major therapeutic objectives in chronic inflammatory rheumatic diseases is the tapering and discontinuation of GCs. However, this process is fraught with challenges, including unpredictable success rates and possible adverse outcomes such as disease flares and adrenal insufficiency. This section evaluates evidence regarding successful withdrawal rates, clinical consequences of tapering, and predictive factors [26-28].

Success Rates of Discontinuation and Predictive Factors

The effectiveness of GC discontinuation is highly variable across patient groups and study designs, suggesting significant challenges. There is a wide variety of success rates observed in observational studies and clinical trials. An example is where the RA cohort on b/tsDMARDs reported only 23% of patients with consistent discontinuation after three years [25]. Discontinuation of GCs in another RA cohort treated with tofacitinib was 30% at 12 weeks [23]. The STAR trial, which included two active tapering interventions in RA, reported discontinuation rates of 47% and 55% at one year [20]. The majority of a cohort of patients diagnosed with SLE shortly before (57.5%) were able to taper and retain a prednisone dose of less than 5 mg/day, and another 13.4% discontinued the medication completely after two years of follow-up [26]. It was determined that several factors are predictors of successful or unsuccessful GC tapering, such as shorter disease duration and younger age. A series of studies on RA cohorts has concluded that successful GC discontinuation was closely associated with a shorter disease course and a younger patient age [24]. However, the age and the duration of the disease were negatively correlated with GCs withdrawal [23]. Moreover, a lower baseline DAS28 was a predictor of GC cessation in patients with RA who began with a b/tsDMARD [25]. In a group of newly diagnosed SLE patients, active renal SLE involvement in the study was potentially an excellent predictor of failure to reduce prednisone to less than 5 mg/day [26].

Withdrawal Effect: Disease Flares

The clinical risk of disease flare following GC withdrawal differs between RA and SLE populations. Recent data show that a gradual taper may lead to better outcomes, particularly in maintaining remission for SLE. For example, in a post-GLORIA trial of RA patients, older patients tapering to prednisolone experienced a higher frequency of flares (45%) compared to those tapering to placebo (33%) [25]. In a similar study, the SEMIRA trial found more RA flares among patients on tapered GCs than those maintained on 5 mg/day of prednisolone [31].

In contrast to RA, SLE cohort studies indicate a protective effect of gradual withdrawal. For example, a randomized trial of patients with non-exacerbating SLE found that gradual withdrawal over several months led to lower flare rates at 24 months compared to continued use of a 5 mg/day dose (33.3% vs. 50.0%) [28]. Similarly, a multinational group of SLE patients in the stable modified serologically active clinically quiescent (mSACQ) stage saw no increased risk of total or severe flares associated with GC reduction [22].

Withdrawal Effect: Adrenal Insufficiency

A significant risk associated with long-term GC therapy is suppression of the HPA axis, which would cause life-threatening adrenal insufficiency on withdrawal. Despite this concern, recent clinical trials have revealed a significant unreported prevalence of adrenal insufficiency. The GLORIA trial did not detect any signs or symptoms of adrenal insufficiency following a 5 mg/day prednisolone taper [25]. Similarly, there were no instances of acute adrenal insufficiency in more than 100 RA patients who received two dissimilar withdrawal plans [20]. Nevertheless, the complete recovery of the adrenal axis does not necessarily come along with the lack of clinical symptoms. The STAR trial used adrenocorticotropic hormone (ACTH) stimulation tests to assess biochemical functioning. The results showed that 17 patients still had abnormal test results 12 months later, thus requiring the continuation of the hydrocortisone replacement test [20-21].

Withdrawal on Damage to Organs

One reason GC should be withdrawn is to prevent permanent, irreversible organ damage. The organ damage among SLE patients is a fundamental outcome measure for tapering GCs. There are findings that tapering is beneficial. A cohort study of patients with quiescent SLE showed that a slow taper of GC was independently associated with less damage at 24 months compared with maintenance treatment (6.9% vs. 17.6%) [29]. This is supported by the GULP study in new SLE, where at least once monthly, a patient who had not received a prednisone dose of less than 5 mg/day was associated with a reduced damage accrual rate [26]. Additional evidence for this is provided by the Asia-Pacific Lupus Collaboration (APLC) cohort, in which tapering GCs was associated with a reduction in damage accrual in mSACQ patients receiving >5 mg/day prednisolone [22]. The positive results of GC tapering not only prevent future adverse effects but also slow the progression of organ damage.

GC Tapering Programs

The way GCs are tapered can significantly affect the outcome of the clinical process, especially the risk of a disease flare. The decision of whether to make it slowly or abruptly, or to employ other replacement methods, has been directly and indirectly compared in recent literature. Also discussed here are the findings of trials conducted that have determined the outcome of various tapering regimes [29-31].

Slow Versus Rapid Discontinuation

The speed of tapering is crucial between rapid and slow. Otherwise, the effect can harm a patient's recovery. There are strong indications that a gradual taper is better than an immediate withdrawal. The randomized study in clinically quiescent SLE patients conducted by Tselios et al. (2021) [29] used a stepwise withdrawal regimen, reducing the daily prednisone dose by 1 mg over a series of months. This gradual taper was also safe and resulted in fewer clinical flares than ongoing maintenance therapy. The authors compared their results with another trial in which the abrupt discontinuation of 5 mg/day prednisone was associated with a high likelihood of a flare within the next 12 months [30]. So, the rate of withdrawal can be considered a predictive factor of immunological development, and gradual tapering may help substantially control the re-equilibration of the immune system compared to sudden termination.

Hydrocortisone Replacement Compared to Regular Prednisone Tapering

The STAR trial was able to make a head-to-head comparison of two different withdrawal strategies in patients experiencing RA in low disease activity with prednisone 5 mg/day [20]. The patients were randomly assigned to either a normal prednisone taper scheme or a hydrocortisone replacement scheme. The main conclusion of the trial was that the hydrocortisone replacement strategy did not prove better than the standard prednisone tapering strategy, with success at 12 months to GC discontinuation (55% vs. 47%, respectively; p = 0.4) [20]. In addition, the two strategies did not show any major differences in secondary outcomes, such as flare rates or patient-reported outcomes.

Implications and Strengths

The findings strongly support the European Alliance of Associations for Rheumatology (EULAR)'s recommendation to taper and discontinue GCs when clinically feasible. GC withdrawal reduces long-term organ damage, especially in SLE, and multi-system adverse effects, making it clinically beneficial despite the risk of illness exacerbation (flare rates). This emphasizes discontinuation as a standard-of-care goal. The results suggest that cautious, slow, gradual tapering is safer and more effective than rapid withdrawal. However, practitioners must consider patient-specific characteristics to maximize discontinuation success rates.

There are the following strengths of this manuscript. The systematic review was conducted following PRISMA guidelines. The methodological quality assessment was performed using standardized Cochrane RoB tools such as ROBINS-I and Cochrane ROB 2.0. The evidence is synthesized using narrative synthesis due to variability in interventions and the tapering approach. The evidence is somewhat substantial, as it is based on moderate- to low-quality RCTs and large multinational observational cohort studies.

Limitations and Future Recommendations

Despite evidence being based on RCTs and observational cohort studies, the quality of evidence in one out of 10 studies is serious RoB, and six studies show moderate or some concerns. Only three out of 10 studies showed low ROB. Therefore, readers must be cautious while interpreting evidence findings. Some studies did not explicitly report withdrawal symptoms. The findings are only applicable to adults because of the selected demographic. However, future researchers are encouraged to conduct more RCTs or clinical trials to validate the findings. The future trials should focus on explicitly mentioning and studying withdrawal symptoms to strengthen the evidence from high- to moderate- to low-ROB studies.

## Conclusions

The evidence supports the EULAR guidelines for tapering and stopping GC use. Although disease flares can occur, long-term organ damage is less likely, especially in SLE, when GC is gradually tapered. These strategies prevent systemic adverse effects and provide clinical benefit to GC withdrawal. Thus, discontinuation remains a cornerstone of standard care. Importantly, the findings favor gradual tapering over sudden withdrawal as safer and more efficient. The clinicians must also consider each patient's individual factors to improve the success of GC withdrawal and tapering.
